# Biomimetic Innovations: Exploring Bubble-Trapping Organisms for Manufacturing Breakthroughs

**DOI:** 10.3390/biomimetics10100641

**Published:** 2025-09-23

**Authors:** Haohan Yu, He Wang, Wei Bing

**Affiliations:** 1 Key Laboratory of Bionic Engineering, Ministry of Education, Jilin University, Changchun 130022, China; 2 School of Chemistry and Life Science, Changchun University of Technology, Changchun 130012, China

**Keywords:** air layer, biomimetic, antifouling, drag reduction, bionic manufacturing

## Abstract

Many aquatic organisms have evolved remarkable micro/nanostructures and surface chemistries that enable stable air bubble entrapment, offering valuable insights for biomimetic engineering. Various fabrication techniques—including chemical deposition, photolithography, 3D printing, electrospinning, electrostatic flocking, and femtosecond laser processing—can replicate these bioinspired bubble-trapping surfaces. Crucially, the optimization of surface physicochemical properties during manufacturing is essential for maintaining stable air layers. These engineered air layers demonstrate dual functionality, serving as both an effective biofouling barrier and a drag-reducing lubricant interface, where bubble characteristics (size, density, and stability) critically determine performance. This review comprehensively examines the biological prototype of bubble adsorption, key physicochemical parameters governing air layer formation, and state-of-the-art biomimetic manufacturing methods. We anticipate that this systematic analysis will advance fundamental understanding of bubble dynamics while inspiring novel applications of air-layer technologies across multiple engineering domains.

## 1. Introduction

The micro/nanostructured superhydrophobic surfaces capable of entrapping air layers have garnered significant research interest due to their multifunctional applications. These underwater air films offer an eco-friendly solution with diverse potential uses, particularly in drag reduction [1] and antifouling [2]. Maintaining long-term stable air layers is the key to realizing its functional application. However, due to the limitations of gravity drainage, environmental disturbance and interface effect, the stability of artificial air layer is still a big challenge [3]. The current research is devoted to addressing this issue through strategies such as bionic structure design, and surface chemical modification. Although the improvement of bubble stability has been demonstrated in laboratory environments through surface optimization, the air layers trapped by most existing artificial superhydrophobic surfaces tend to gradually undergo a Wenzel state transition and eventually fail when submerged underwater, particularly under conditions such as hydrostatic pressure [4], and fluid shear stress [5]. This failure is mainly attributed to the deficiencies of artificial surfaces in terms of structural mechanical strength, morphological accuracy, and control of chemical heterogeneity. These limitations prevent them from maintaining a stable solid–liquid–gas three-phase contact line dynamically as in biological systems.

In contrast, biological structures that have evolved over hundreds of millions of years (such as the egg-beater-shaped trichomes of *Salvinia*) offer naturally validated optimized solutions to this stability dilemma. This is achieved through their intricate hierarchical structures, mechanical properties balancing rigidity and flexibility, and ingenious hydrophilic-hydrophobic patterning [6]. Therefore, there is an urgent need to disassemble the intrinsic stability mechanisms of these biological prototypes, as this will provide critical guidance for the design of next generation of durable biomimetic air-layer-retaining surfaces.

Through evolutionary adaptation to aquatic environments, numerous organisms have developed sophisticated bubble-entrapment mechanisms, such as lotus leaves, *Salvinia*, water spiders, etc. The lotus leaf employs a hierarchical micropapillary architecture that creates stable underwater air layers by trapping gas within its surface topography [7]. *Salvinia* species feature specialized egg-beater-shaped trichomes with dual wettability: superhydrophobic shafts for air retention and hydrophilic tips that anchor the air–water interface through the “pinning effect” [8,9]. Similarly, water spiders utilize dense arrays of hydrophobic abdominal hairs to form persistent air pockets that function as respiratory organs [10]. These biological systems demonstrate remarkably efficient air-trapping capabilities through optimized surface morphologies and wetting properties. Such natural designs have inspired emerging biomimetic research focused on developing durable air-retaining materials, representing a promising frontier in surface engineering and functional material development.

**Figure 1:**
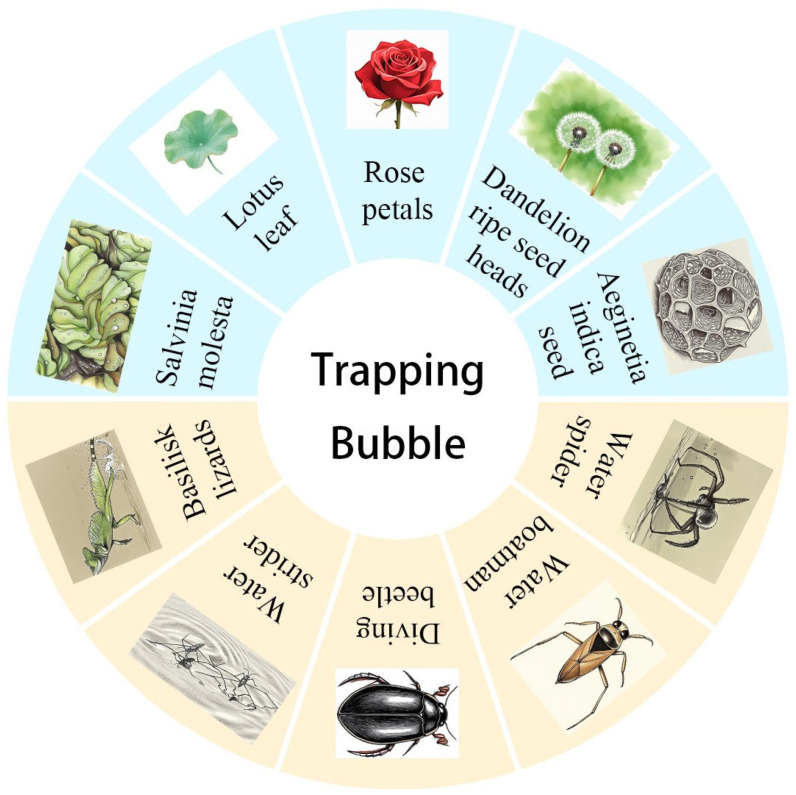
Natural creatures capable of trapping air bubbles.

This review provides a comprehensive synthesis of air-layer-forming organisms in nature, encompassing diverse animal species, plant structures, and specialized biological architectures (Figure 1). These natural prototypes offer rich inspiration for developing bubble-trapping materials through various biomimetic fabrication approaches, such as chemical deposition [11], photolithography [12], 3D printing [13], electrospinning [14], electrostatic flocking [15], femtosecond laser [16], etc. However, there are still many problems, such as the inability of large-scale preparation and the instability of air layer. As an environmentally friendly strategy, the air layer can be applied in various fields, especially in antifouling and drag reduction. Herein, we summarize the physicochemical properties of natural structures that can capture bubbles, explore the factors that affect the stability of air layer, and focus on its application in antifouling and drag reduction. We expect that the present review can attract researchers from bionics, material science, physical chemistry and other disciplines to further optimize the properties of bionic functional materials that can capture bubbles, and develop new bionic materials that can maintain the air layer stable for a long time.

## 2. The Theoretical Basis for the Stability of Underwater Air-Layers

The remarkable ability of biological surfaces to trap and stabilize air layers underwater is not only a novelty but also a direct consequence of well-defined physicochemical principles. This section systematically clarifies the core theoretical framework that underpinned the origin and stability of these air layers, laying a predictive foundation for understanding natural phenomena and their bionic applications. Mastering these control principles is crucial for designing functional surfaces with customized characteristics, including anti-fouling performance, drag reduction capability, etc.

### 2.1. Wettability and the Contact Angle

The water-repellent property of a surface is quantifiably expressed by its contact angle, which is defined as the angle at the solid–liquid–gas interface. For an ideal, smooth, and chemically homogeneous surface, the equilibrium contact angle is given by Young’s equation [17]:

(1) $$\gamma_{\mathrm{sv}}=\gamma_{\mathrm{sl}}+\gamma_{\mathrm{lv}}\cos\theta$$

where *γ*_sv_, *γ*_sl_, and *γ*_lv_ represent the solid–vapor, solid–liquid, and liquid–vapor interfacial tensions, respectively. *θ* is the angle between the tangent line of the water drop surface and the horizontal plane. When water droplets adopt a spherical morphology with a contact angle exceeding 150° on a solid surface, the material demonstrates superhydrophobicity. This exceptional surface property emerges from the synergistic effect of low surface energy combined with micro/nanoscale surface roughness. Superhydrophobicity is the fundamental prerequisite for the formation of a persistent air layer upon the solid surface immersed in water. When superhydrophobic surfaces are submerged in water, their micro/nanostructures can entrap air, forming stable air layers at the solid–liquid interface. Importantly, the contact state between the substrate and the liquid medium plays a decisive role in determining the stability of bubbles anchored to nanoscale substrates [18].

The apparent contact angle (*θ*_w_) on a rough surface is governed by one of two primary models. The Wenzel model [19] describes the homogeneous wetting state where liquid completely penetrates the surface grooves. The apparent contact angle is given by

(2) $$\cos\theta_{w}=r\cos\theta$$

In this equation, *r* is the surface roughness factor (*r* > 1 for a rough surface), and *θ* is the intrinsic Young’s contact angle. The model shows that surface roughness results in a larger apparent contact angle on hydrophobic materials.

On rough surfaces, the apparent contact angle is more accurately described by the Cassie–Baxter model [20], which accounts for the air trapped within the surface textures. The establishment and maintenance of this metastable Cassie state are the fundamental basis for the formation of a persistent air layer. The apparent contact angle is given by

(3) $$\cos\theta_{\mathrm{cb}}=f_{s}\cos\theta +f_{s}-1$$

where *f*_s_ represents the fractional area of the solid in contact with the liquid. When the solid fraction *f*_s_ is very small, a very high apparent contact angle (approaching 180°) can be achieved even with a moderately hydrophobic material (*θ* > 90°).

The formation and maintenance of the Cassie–Baxter state are essential for sustaining a stable air layer after immersed in water. When a superhydrophobic surfaces are submerged in water, micro/nanostructured surfaces entrapped air to establish a stable air layer at the solid–liquid interface. The contact state between the substrate and the liquid medium plays a decisive role in the stability of bubbles anchored to nanoscale substrates [20]. Importantly, the transition from the Cassie–Baxter state to the Wenzel state is usually induced by external factors such as pressure or vibration, which can lead to irreversible loss of the air layer and the collapse of superhydrophobicity.

### 2.2. Hydraulic Pressure

The key variable of water pressure affects the stability of air layer. Sheng et al. [21] studied the effect of pressure on the stability of the air layer by applying external pressure to the surface of lotus leaves. Research has found that when the pressure exceeds 0 kPa and remains below the critical collapse pressure (critical pressure, Pc ≈ 13.5 kPa), the air layer on the lotus surface remains stable [21]. Under this condition, the water pressure can prevent the dissolution and diffusion of air. According to Henry’s Law, the solubility of a gas in a liquid is directly proportional to its partial pressure. When a superhydrophobic surface is immersed in water, the air trapped in the microstructures will continuously dissolve and diffuse into the surrounding water. Applying appropriate additional water pressure increases the environmental pressure of the air layer, which elevates the saturated solubility of air in water, thereby retarding the dissolution and diffusion of trapped air. In this state, the slow dissolution rate minimizes air layer depletion, enhancing its stability. Essentially, within the optimal pressure range (0 < P < Pc), both air dissolution is slowed and air is forced deeper into microstructures, resulting in a more stable water layer compared with ambient pressure conditions. However, excessive pressure (P ≥ Pc) disrupts the liquid–air interface, allowing water to invade micro/nanostructure voids. This triggers the transition from the Cassie state to complete wetting Wenzel state, causing instantaneous air layer collapse.

Therefore, maintaining an appropriate water pressure is crucial for the stability of the air layer on the underwater superhydrophobic surface, which may be of great significance for achieving low resistance or anti-friction effects on superhydrophobic surfaces.

### 2.3. Buoyancy

The stability of the air layer is significantly challenged by buoyancy. As a primary destabilizing force, buoyancy promotes the coalescence and upward migration of trapped air pockets, ultimately leading to their detachment from the surface and the collapse of the air layer.

A fundamental stability criterion is governed by the balance between the buoyant force (*F*_b_) acting to detach a bubble, and the capillary force (*F*_c_) that anchors it to the surface. For a spherical bubble of radius R, these forces can be expressed as follows:

(4) $$F_{b}=\frac{4}{3}{\pi R}^{3}\rho_{w}g$$

(5) $$F_{c},\max\approx 2\pi R_{C}\gamma \left(\cos\theta_{r}-\cos\theta_{a}\right)$$

where *ρ*_w_ is the density of water, *g* is gravity, *R*_c_ is the radius of the three-phase contact line, *γ* is the liquid surface tension, *θ*_r_ and *θ*_a_ are the receded and advanced contact angles. To maintain the stability of the air bubbles, it is necessary to keep *F*_c_ greater than *F*_b_. This force balance reveals that smaller bubbles (small R) are more capable of maintaining stability.

However, in practical scenarios involving microstructured surfaces, the failure mechanism is more complex, typically involving the thinning and rupture of the wetting film—the thin liquid layer separating the bubble from the solid nanostructures—which plays a critical role as buoyancy attempts to detach the bubble [22]. This effect is particularly pronounced on inverted superhydrophobic surfaces [22,23].

The stability of a bubble under these conditions can be more precisely analyzed by considering the Laplace pressure difference across the air–water interface trapped within surface features such as re-entrant cavities. For capillary structures with height-dependent cross-sections, this pressure at the three-phase contact line can be approximated by the following equation [24,25]:

(6) $$P_{g}-P_{o}=\rho gH-\frac{2\gamma \cos\left(\phi +\alpha \right)}{R+h\tan\alpha }$$

where *P*_g_ and *P*_o_ are the pressures of the captive air pocket and the atmosphere, respectively, *ρ* is the density of water, *g* is the gravitational acceleration, *H* is the depth below the waterline, *γ* is the liquid–gas surface tension, *ϕ* is the equilibrium contact angle, *α* is the angle between the sidewall of the solid structure and the vertical direction, *R* is the principal radius of curvature of the gas–liquid interface in the horizontal plane at the three-phase contact line, and *h* is the vertical height from the gas–liquid interface to the solid reference plane.

A positive Laplace pressure (*P*_g_ > *P*_o_), often achieved through optimizing *α* and *ϕ* in re-entrant structures, establishes an energy barrier that counteracts both external hydrostatic pressure and wetting film drainage, thereby enhancing stability against buoyancy-driven collapse. Conversely, if the geometry leads to a negative Laplace pressure, it can accelerate the drainage and rupture of the film, facilitating bubble coalescence and the formation of gas bridges, ultimately causing air layer failure. Therefore, although buoyancy is a decisive environmental factor, active suppression through surface microstructure design can ensure continuous air layer stability.

### 2.4. Surface Structure

The design of surface microstructure provides necessary countermeasures to enhance air layer stability. Multi-level microstructures can enhance the stability of air layer by increasing the gas content in grooves, thereby prolonging the diffusion time of gas into water [26]. The hierarchical architecture creates additional liquid–gas pinning sites during wetting transitions, elevating the activation energy barrier and consequently enhancing air layer stability [27]. Just like the microstructure on the surface of springtails, processing the microstructure into a concave feature is conducive to pinning the liquid–gas interface at the microstructure edge, thereby enhancing the stability of air layer [28,29]. Therefore, the stability of air layer can be achieved by using low surface energy substances or through chemical modification, and obtain superhydrophobic multi-level micro/nano surfaces [30,31].

### 2.5. Advanced Stabilization Mechanisms in Biological Systems

Nature has evolved sophisticated strategies, beyond the basic Cassie state, to enhance the air layer stability. Structures featuring overhangs or inward curvatures (e.g., mushroom-like or T-shaped architectures) mechanically anchor the air–water interface. This design enables stable operation even on substrates with moderate hydrophobicity while demonstrating remarkable pressure resistance.

These are geometries that possess an overhang or inward curvature (such as mushroom-like, T-shaped). They geometrically lock the air–water interface, enabling stability even on surfaces with lower intrinsic hydrophobicity and providing exceptional resistance to pressure.

The elasticity of microstructures (such as the hairs of *Salvinia*) provides a dynamic defense mechanism. Under pressure, elastic structures bend rather than allow water infiltration, dissipating energy and preventing the collapse of the air–water interface. After the pressure is released, they exhibit sufficient elastic recovery and quickly rebuild a stable air layer.

In addition to passive retention, biological systems can also exploit fluid dynamics to actively replenish gas. Cavitation, the nucleation of vapor bubbles due to local pressure drops in flowing liquids, serves as a potential active gas source. This phenomenon can be harnessed in biomimetic design. For instance, Gonzalez-Avila et al. [32] demonstrated that micro/nanostructured surfaces can stabilize cavitation bubbles, forming a continuous air layer that significantly reduces drag under flow conditions. By designing surfaces to promote and stabilize localized cavitation, a passive, decaying air layer can be transformed into an active, self-replenishing system, overcoming a key limitation for long-term applications.

### 2.6. Design Principles

The stability of an underwater air layer is an interplay of surface chemistry, morphology, and mechanical forces. The transition from descriptive observation to quantitative design requires adherence to the following principles, summarized in Table 1.

This theoretical framework provides the necessary tools for analyzing the biological structures presented in the following section and for guiding the rational design of advanced biomimetic surfaces, moving beyond mere morphological imitation to functional replication based on first principles.

**Table 1:** Design principles for biomimetic air-retaining surfaces.

Design Objective	Underlying Principle	Key Design Parameters	Biomimetic Example	Target Application	Refs
Maximize air entrapment	Achieve and maintain Cassie–Baxter state	Contact angle;	Lotus leaf micro/nanopapillae	Superhydrophobicity, self-cleaning	[33]
		Solid fraction;			
		Hierarchical roughness			
Enhance stability under underwater pressure	*Salvinia* Effect, dual Cassie–Baxter state	Number and size of head filaments	Foliar hair structure of *Salvinia molesta*	Underwater equipment, low-energy transportation	[34]
Anchor the air–water interface	The lotus effect, low surface energy	Micro/nano composite structure;	The lotus microstructure	Stable plastron for antifouling	[35]
		High contact angle, low sliding angle			
Enable dynamic response	Utilize structural elasticity	Low modulus;	Elastic hairs of *Salvinia*	Energy absorbing material	[36]
		High elasticity			
Active gas injection	Superhydrophobic surface	Porous nested structure	Lotus leaf, water strider leg	Anti-icing, drag reduction	[37]

## 3. Natural Structures

### 3.1. Plant Structures

Nature provides numerous examples of both aquatic and terrestrial plants capable of capturing and maintaining stable air layers or bubbles underwater [24,38,39,40,41,42]. The surfaces of these unique plants all have unique micro/nanostructures, which can trap air in the surface topography, such as *Salvinia* [9], lotus leaves [43], rose petals [40] and dandelion ripe seed heads [41]. We analyzed and summarized the physicochemical properties and structural factors that can affect the formation and stability of air layer. In biomimetic surface fabrication, the extraction and implementation of these critical biological design principles represent a crucial step toward developing functional materials with superior bubble-trapping capabilities. Consequently, comprehensive investigation of bubble stabilization mechanisms in biological prototypes remains essential for advancing biomimetic manufacturing technologies.

#### 3.1.1. *Salvinia molesta*

**Figure 2:**
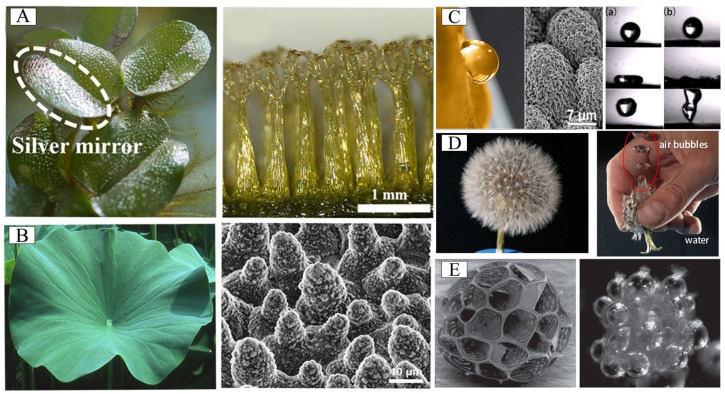
(**A**) The superhydrophobic leaves of *Salvinia molesta* are covered with egg beater-like structures. Adapted with permission from Ref. [9]. Copyright 2025 Elsevier Ltd. (**B**) The surface of the lotus leaf is composed of papillary micro/nanostructures. Adapted with permission from Ref. [61]. Copyright 2011 Beilstein-Institut. (**C**) Droplet impact dynamics of yellow rose petals: (**a**) Impact at low kinetic energy (release height h = 8 mm); (**b**) Impact at high kinetic energy (h = 150 mm). Adapted with permission from Ref. [56]. Copyright 2023 American Chemical Society. (**D**) The dandelion ripe seed heads are immersed in water, and bubbles can be seen after squeezing the seed heads. Reprinted with permission from Ref. [41]. Copyright 2021 Elsevier Ltd. (**E**) SEM images of *Aeginetia indica* seeds under drying and soaking conditions. Reprinted with permission from Ref. [60]. Copyright 2016 The Royal Society.

*Salvinia molesta*, a free-floating aquatic fern, exhibits remarkable leaf surface morphology characterized by numerous eggbeater-shaped structures (Figure 2A) [9]. Each of these distinctive structures comprises four vertically aligned hairs that converge at their apex, forming a unique architectural configuration. The terminal region of these structures features hydrophilic patches, while the remaining surfaces are coated with hydrophobic wax crystals [44,45]. This sophisticated heterostructure endows *Salvinia* leaves with exceptional functional properties. Specifically, the hydrophilic apical regions serve to anchor the air–water interface, thereby stabilizing the air layer retained within the hydrophobic zones, which is called the “*Salvinia* Effect” [44,46,47,48].

#### 3.1.2. Lotus Leaf

The microstructural features of lotus leaves are illustrated in Figure 2B, which exhibits a hierarchical architecture comprising epidermal wax and uniformly distributed micropapillae. These micropapillae are further decorated with dendritic nanostructures, collectively conferring remarkable superhydrophobicity and reduced surface adhesion. This unique surface topography enables the well-documented self-cleaning capability. The synergistic effect of hydrophobic wax coating and micro/nanostructures results in extreme water repellency, with contact angles consistently exceeding 150° and sliding angles below 5°. This phenomenon, known as the “lotus effect”, facilitates spontaneous water droplet removal from the surface [21,49,50,51,52]. Furthermore, the sophisticated surface morphology promotes air entrapment, leading to the formation of discontinuous three-phase contact lines at the solid–liquid–air interface [53,54].

#### 3.1.3. Rose Petals

The surface morphology of rose petals exhibits a unique hierarchical structure consisting of micropillars and nanofolds, endowing them with distinctive superhydrophobic characteristics (Figure 2C). Compared with general superhydrophobic surfaces, rose petals have high adhesion to water droplets (“petals effect”) [55] and the ability to capture bubbles underwater (stable “pinning effect”), as shown in Figure 2C. These phenomena originate from the petal’s sophisticated surface architecture, where the delicate yet rough nanoscale protrusions effectively trap air layers when submerged. When air bubbles approach the submerged petal surface, significant contact angle hysteresis occurs, resulting in bubble adhesion to the surface. Crucially, these functional capabilities are fundamentally governed by the inter-papillae spacing within the petal microstructure.

Furthermore, studies have demonstrated that the stability of the gas film is significantly influenced by both the volume and directional flow of trapped gas beneath the droplet [56]. Compared with the shorter micro-domed layered structure on rose petals, the high columnar layered structure on lotus leaves can accommodate a greater amount of air and provide the most stable air film under the droplets. In contrast, the petals of red roses have typical sticky superhydrophobic surfaces that remain sticky under any circumstances, while the petals of yellow roses exhibit variable wettability. This is mainly due to the change in the stability of the air film trapped beneath the impact droplet and the surface of the target solid. Notably, the isotropic nanofolds on yellow rose petals demonstrate a distinctive dynamic wetting transition upon droplet impact: exhibiting complete rebound behavior at low impact energy (h ≤ 20 mm) but shifting to micro-pinning state at elevated energy levels (h ≥ 50 mm) (Figure 2C) [56]. This dynamic response stands in marked contrast to their static high-adhesion characteristics.

#### 3.1.4. Dandelion Ripe Seed Heads

The mature seed heads of dandelions exhibit a remarkable morphological adaptation, featuring individual seeds equipped with pappus parachute structures. When submerged in aqueous environments, the umbrellas at the top of each pappus encase the droplets, so that all the wet umbrellas formed a barrier that helped the seed heads retain air (Figure 2D) [41,57,58,59]. Surprisingly, this air-holding behavior is fully or partially reversible when submerged again in water, and this behavior also generates an upward thrust [41].

#### 3.1.5. *Aeginetia indica* Seed

The seeds of *Aeginetia indica* achieve efficient air capture and underwater air layer stability through a unique micro-compartmentalized grid structure. It features a multi-level grid structure, with the seed epidermal cell wall locally thickened by cellulose, forming a rigid grid frame (Figure 2E) that divides the surface into hundreds of independent micro-compartments [60]. This makes the seeds of *Aeginetia indica* have a dual-mode propagation strategy. In the air, its lightweight grid structure optimizes the efficiency of wind-borne propagation. When encountering water, the instantaneous buoyancy generated by bubbles realizes hydraulic transmission and expands the ecological niche.

### 3.2. Natural Animal

Beyond plant structures capable of trapping air layers, numerous animal species have also evolved mechanisms to capture and maintain air bubbles, forming protective air layers. Notable examples include aquatic insects like water boatmen, diving beetles, and water striders, as well as vertebrates such as *Basilisk lizards* and carp. These surface air layers serve vital biological functions, primarily facilitating respiration or enabling rapid locomotion across water surfaces.

#### 3.2.1. Water Spider

**Figure 3:**
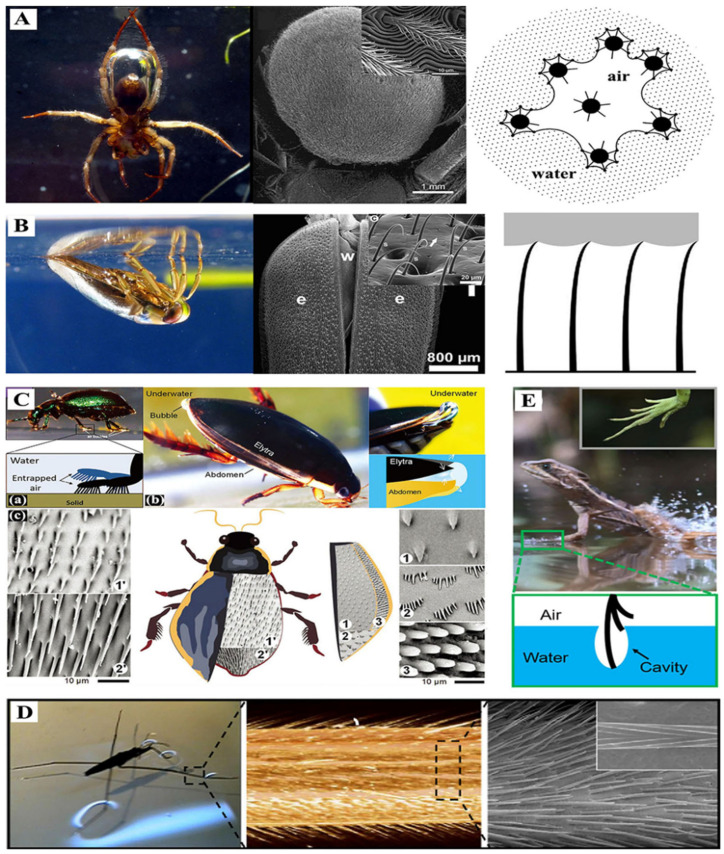
(**A**) The feathery hairs on the abdomen of *A. aquatica* anchor air bubbles underwater. Adapted with permission from Refs. [38,67]. Copyright 2011 The Company of Biologists; 2013 BioMed Central Ltd. (**B**) The curved hairs of *N. glauca* on its wings that lead to the formation of a silvery air layer underwater. Adapted with permission from Refs. [80,81]. Copyright 2011 Beilstein-Institut. (**C**) (**a**) *G. viridula’s* feet trapped air bubbles underwater. (**b**) The air bubbles attached between the elytra and abdomen of *C. tripunctatus*. (**c**) Body parts images of *C. tripunctatus.* Adapted with permission from Refs. [69,72,82]. Copyright 2021 Wiley; 2012 The Royal Society; 2023 the Royal Society. (**D**) The bristle structures of *G. remigis* on the legs that allow the formation of an air layer between the legs and the water surface. Adapted with permission from Ref. [83]. Copyright 2015 National Academy of Sciences. (**E**) *Basilisk lizards* striking and deforming the free water surface to form an air-entrained cavity by utilizing their cylindrical toes. Adapted with permission from Ref. [84]. Copyright 2021 American Chemical Society.

The water spider *Argyroneta aquatica* (*A. aquatica*) represents a remarkable example of permanent underwater adaptation among arachnids [62]. This species possesses specialized morphological adaptations, including hydrophobic hairs covering its abdomen and legs that create an air-trapping surface, enabling the formation of stable abdominal air bubbles (Figure 3A) [38]. These bubble structures function as “physical gills” through their gas exchange capabilities, providing essential oxygen for the spider’s submerged existence [63,64]. Furthermore, *A. aquatica* exhibits unique behavioral adaptations by constructing submerged “diving bells” using silk anchored to aquatic vegetation, which serve as additional air reservoirs [38]. This combination of morphological and behavioral adaptations allows these spiders to remain submerged for extended periods, often lasting several days [65].

#### 3.2.2. Water Boatman

The aquatic insect *Notonecta glauca* (*N. glauca*), commonly known as the water boatman, possesses a specialized wing structure comprising two pairs of wings—protective forewings and functional hindwings that remain concealed beneath them. This species exhibits a unique respiratory behavior, periodically surfacing to replenish its air supply before submerging again. During submersion, the hindwings’ surface morphology, characterized by numerous curved micro-hairs, facilitates the formation of a distinctive silvery air bubble layer (Figure 3B) [66,67,68]. This trapped air film serves as both a respiratory reserve and buoyancy regulator during the insect’s underwater activities.

#### 3.2.3. Diving Beetle

The terrestrial leaf beetle *Gastrophysa viridula* demonstrates remarkable underwater locomotion capabilities through specialized tarsal structures. Its sticky bristles effectively trap air bubbles, enabling stable underwater walking (Figure 3C(a)) [69,70,71]. Similarly, the diving beetle *Cybister tripunctatus* exhibits sophisticated adaptations, with its sheath and abdomen forming oxygen-exchanging air bubbles that sustain underwater respiration (Figure 3C(b,c)) [72]. Notably, the beetle’s elastic abdominal interface allows dynamic bubble manipulation—through separation and compression, it creates concave-convex bubble morphologies that enhance adhesion strength [72]. These adaptations, collectively termed “physical gills”, enable certain diving beetle species to remain submerged for weeks via efficient gas exchange mechanisms [63,73].

#### 3.2.4. Water Strider

The water strider *Gerris remiges* exhibits remarkable aquatic locomotion capabilities, effortlessly standing on water surfaces and achieving rapid movement speeds up to 1.5 m/s [74]. This exceptional performance stems from the insect’s specialized leg morphology, which features hierarchical micro/nanostructures including needle-like bristles with precisely arranged nano-grooves (Figure 3D) [75]. These intricate structures create a superhydrophobic surface with a contact angle exceeding 150°, enabling the legs to repel water effectively. The combined effects of surface tension (providing buoyancy) and extreme water-repellency allow the water strider to maintain stable flotation, with microscopic observations revealing a persistent air layer (plastron) at the leg-water interface (Figure 3D) [75,76]. This air layer has multiple functions, including reducing resistance during locomotion, providing additional buoyancy, and preventing wetting that could compromise mobility [77]. Furthermore, the air plastron facilitates efficient momentum transfer during the insect’s characteristic rowing motion, explaining its ability to execute rapid directional changes and escape maneuvers.

#### 3.2.5. *Basilisk lizards*

The remarkable cylindrical toes of *Basilisk lizards* present groundbreaking biomechanical insights that could revolutionize our understanding of fluid dynamics and locomotion. These extraordinary reptiles achieve this feat through a sophisticated interaction between their uniquely shaped toes and the water surface. As depicted in Figure 3E, when the lizard cylindrical toes strike the water, they deform and expand the air–water interface, generating temporary air cavities between the toes and the water surface [78]. This phenomenon creates a dynamic system where the formation and collapse of these air cavities produce upward momentum [79], effectively counteracting gravity and allowing the lizard to maintain buoyancy during its rapid movement across the water. The study of this mechanism not only enhances our comprehension of animal locomotion but also inspires potential applications in biomimetic robotics and novel propulsion systems for aquatic vehicles. Future research could explore how variations in toe morphology and impact velocity affect the stability and efficiency of this remarkable water-running capability.

## 4. Artificial Biomimetic Air Layer Structure

The diverse biological paradigms of underwater air-layer retention outlined in Section 3 provide a foundational blueprint for engineered biomimetic surfaces. Based on these natural designs, researchers have developed a variety of artificial surfaces with air retention capabilities through techniques including chemical vapor deposition, lithography, electrospinning, and electrostatic methods. Alternative approaches involve spraying hydrophobic coatings or creating specialized microstructures such as microcavities. These biomimetic surfaces are engineered by replicating the morphological and physicochemical features of natural structures, thereby endowing the artificial surfaces with bubble-trapping properties. The subsequent sections systematically delineate these preparation processes and engineered surface architectures, which purposefully mirror the biological paradigms established in the preceding section. To systematically summarize the transition from biological inspiration to functional artificial surfaces, we have compiled the key biological features, biomimetic design principles, and potential applications in Table 2. This framework serves as a concise guide for researchers aiming to design bubble-trapping surfaces based on natural models.

**Table 2:** Bionic prototypes, design principles and applications from biological prototypes to artificial surfaces.

Biological Prototype	Key Biological Feature	Biomimetic Design Principle	Example Applications	Refs.
*Salvinia molesta*	Egg-beater-shaped hairs with hydrophilic tips	Imitate microstructure to stabilize air layer	Antifouling coatings	[2]
Water spider	Micron diameter, conical structure	Utilizing Laplace pressure difference, directional transport of driving gas	Drag reduction, anti-corrosion, physical gills equipment.	[85]
Lotus leaf	Microstructure of papillae of lotus leaf	Bionic structure, chemical modification	Self-cleaning, anti-icing	[86]
Water bug	Micron bristle array on the surface of insect wings	High contact angle, low sliding angle and high hydrostatic pressure resistance.	Extreme environmental protection	[87]
Diving beetle	The superhydrophobic abdomen of insects can capture and hold bubbles stably.	Morphological control strategy	Capture, fix and spread ozone bubbles	[88]
Water strider	Micron bristle diameter and nano groove depth	Nanometer roughness follows Cassie–Baxter model.	Bioinspired cargo carriers, oil/water separation	[89]
Rose petal	Micropillars with nanofolds (“petal effect”)	Adjust the ratio of Cassie state (low adhesion) and Wenzel state (high adhesion).	Directional diffusion	[90]
*Basilisk lizard*	Hit water to form a cavity, reduce contact area and reduce friction resistance.	Cavity capture and drag reduction	Drag reduction, deep diving	[84]
Carp scale	Orderly arranged micro/nano papillae	Super hydrophilic surface, water will completely soak its rough structure.	Intercept and collect bubbles	[55]

### 4.1. Salvinia molesta-Inspired Air Layer Surface

**Figure 4:**
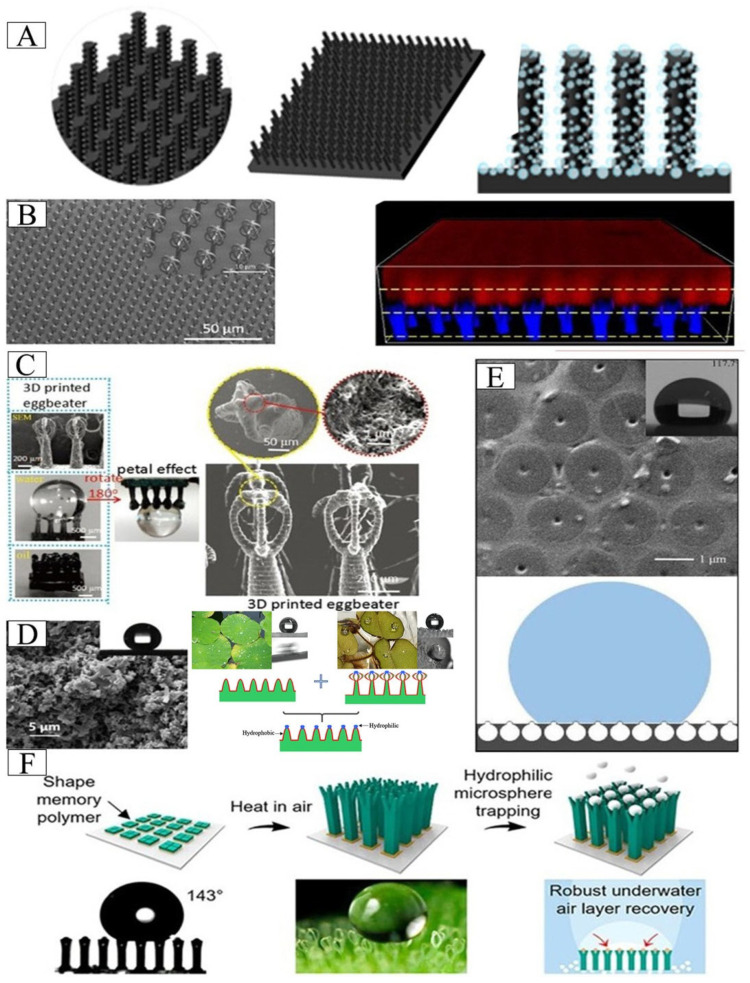
(**A**) The bubble layer is used as the physical anti-fouling barrier to avoid biofouling. Adapted with permission from Ref. [2]. Copyright 2024 Elsevier Ltd. (**B**) Direct laser lithography creates *Salvinia* bionic eggbeater structure that traps air layers underwater (water is colored in red, the structures in blue, air in black). Adapted with permission from Ref. [91]. Copyright 2023 Wiley. (**C**) The bionic structure of the 3D printed egg-beater achieves the “*Salvinia* Effect”, which can form an air layer. Adapted with permission from Ref. [13]. Copyright 2017 Wiley. (**D**) Cassie state biomimetic surfaces were fabricated from dopamine polymers with hydrophilic tips and superhydrophobic microstructures with trapped air layers. Adapted with permission from Ref. [94]. Copyright 2020 Elsevier Ltd. (**E**) Silicone coating prepares the surface of microcavity arrays that can trap air. Adapted with permission from Ref. [96]. Copyright 2022 Elsevier Ltd. (**F**) A *Salvinia* biomimetic structure was prepared by combining shape memory polymer and hydrophilic SiO_2_ microspheres to capture the air layer. Reprinted with permission from Ref. [97]. Copyright 2022 American Chemical Society.

Inspired by the eggbeater-shaped hairs of *Salvinia molesta*, a biomimetic surface was fabricated by combining silicone rubber, graphene, and hydrophobic silica nanoparticles. This design features a micrometer-scale cone array that stabilizes a macroscopic gaseous plastron underwater, providing a physical antifouling barrier through bubble shielding. (Figure 4A) [2]. Direct laser lithography technology can achieve higher-precision structural replication, as shown in Figure 4B. Research shows that even miniaturized bionic structures can effectively capture air, with a retention time of over 100 h [91,92]. In addition, the cellulose acetate nanofiber/micropattern composite surface prepared by combining photolithography and electrospinning techniques, as well as the bionic *Salvinia* microcolumn structure prepared by photolithography and spontaneous adsorption, all demonstrated excellent air retention performance [91]. Inspired by *Salvinia* leaves, Shi et al. [93] designed a simple method to make sticky *Salvinia*-like microcolumns by photolithography and spontaneous adsorption of organic molecules from the atmosphere. With the continuous adsorption of hydrocarbons on the sputtered CeO_2_ film, the surface gradually changed from superhydrophilic to superhydrophobic. Both electrostatic flocking technology and 3D printing technology can obtain biomimetic surfaces that can retain air pockets [13,15]. Among them, the 3D printing structure realizes the precise egg beater structure, and in addition to the performance of capturing air, it also has a pinning effect on water (Figure 4C). These bionic surfaces have significant application prospects in fields such as drag reduction coatings, anti-biofouling, and microfluidic control. Especially the precise “egg beater” structure achieved by 3D printing technology not only perfectly simulates *Salvinia*’s air capture mechanism but also introduces a unique water pinning effect, providing a new idea for the development of the next generation of functional interface materials.

Building upon *Salvinia*-inspired research, several innovative approaches have been developed for creating biomimetic surfaces. A fluorine-free superhydrophobic coating by combining metal silicates (such as cobalt, copper, iron and nickel variants) with nano-silica, nano-titania and aluminum stearate, achieving pinning effect and gas retention performance [47]. Polymerizing dopamine (PDA) onto micro/nanostructured superhydrophobic surfaces renders the tips hydrophilic while conferring the ability to trap air and enhance robust stability (Figure 4D). This modification leverages the strong adhesion of PDA to improve interfacial bonding and leveraging hydrophilic tips to achieve targeted air-trapping functionality, collectively contributing to the observed stability enhancement [94]. Smyrnakis et al. [95] obtained a superhydrophobic plasma micro/nano-textured surface and used white light reflectance spectroscopy for the first time to monitor the surface in real time. They found that its underwater superhydrophobicity and air layer were maintained for at least 60 days. Further advancements include microcavity arrays on silicone-coated surfaces (Figure 4E) that generate exceptionally stable underwater bubble arrays [96], and innovative heterogeneous four-branched microstructures combining shape-memory polymers with hydrophilic silica microspheres (Figure 4F), which exhibit superior air layer retention and self-repair capabilities under both negative and positive pressure conditions [97].

### 4.2. Water Spider-Inspired Air Layer Surface

**Figure 5:**
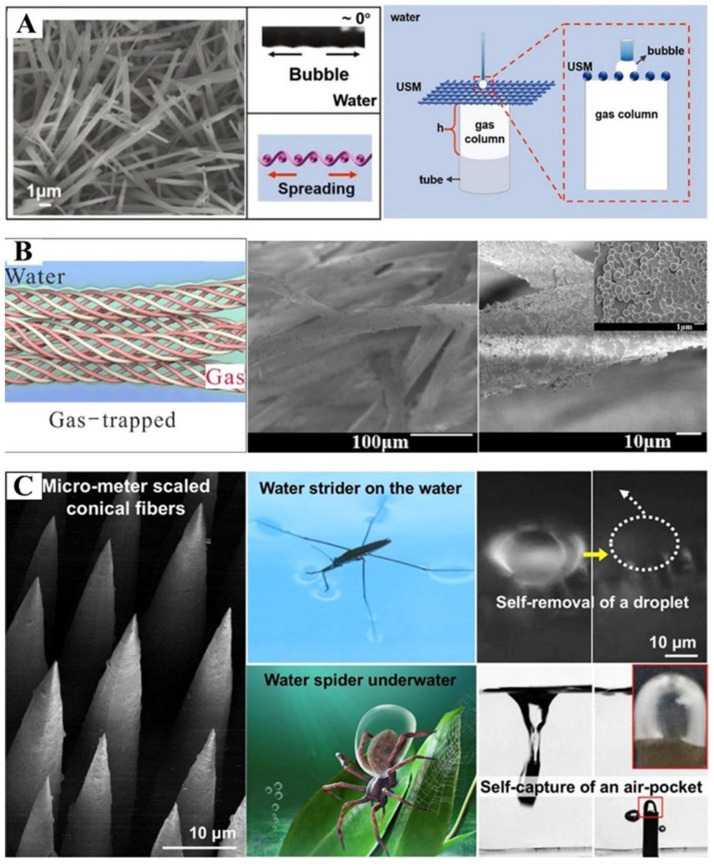
(**A**) USM collects transport bubbles underwater. Adapted with permission from Ref. [98]. Copyright 2019 Wiley. (**B**) SEM image of yarn and schematic diagram of adsorbed air bubbles. Adapted with permission from Ref. [99]. Copyright 2020 American Chemical Society. (**C**) Preparation of M-CFA based on water spider and water strider to capture air pockets. Reprinted with permission from Ref. [85]. Copyright 2022 American Chemical Society.

Inspired by the “diving bell” of water spider, Ning et al. [98] developed a novel single-layer underwater superaerophobic mesh (USM) that effectively collects and transports gas bubbles through an integrated quartz tube system (Figure 5A). Zhang et al. [99] drew inspiration from the unique air-trapping properties of *Argyroneta* spider silk, which maintains stable air layers through its specialized fiber morphology. Their bioinspired superhydrophobic yarn exhibits a distinctive dual-scale roughness (microscale fibers with nanoscale protrusions) that reduces surface energy, enabling exceptional bubble entrapment efficiency (Figure 5B) and controlled directional transport in aqueous environments. Inspired by the structural similarities between Gerris legs and *Argyroneta* abdominal morphology, Tang et al. [85] developed a superhydrophobic microscale conical fibers array (M-CFA). M-CFA was able to capture large air pockets underwater (Figure 5C), with the entrapped air pockets maintaining stability for an impressive duration of 41 days.

### 4.3. Lotus Leaf-Inspired Air Layer Surface

**Figure 6:**
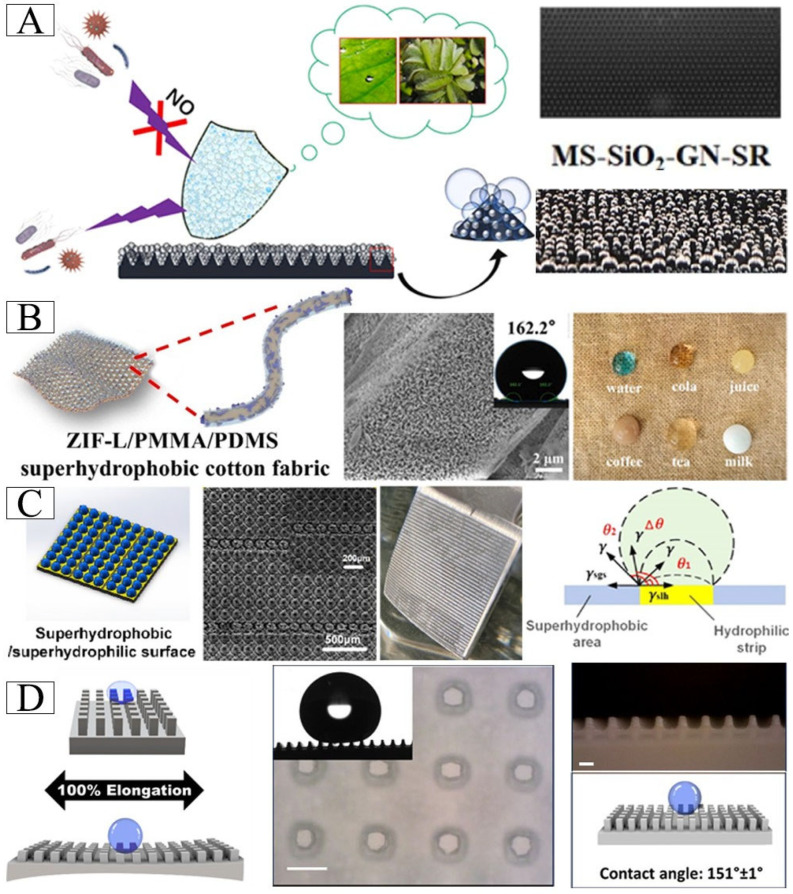
(**A**) Biomimetic lotus leaf hierarchical structure, MS-SiO_2_-GN-SR composite film was developed. The macro air shield formed by it can physically isolate microbial adhesion. Adapted with permission from Ref. [43]. Copyright 2024 Elsevier Ltd. (**B**) The superhydrophobic cotton fabric has antifouling performance. Adapted with permission from Ref. [86]. Copyright 2025 Elsevier Ltd. (**C**) Multiscale apparent structure of BSHSs and schematic of the stress balance condition of the three-phase contact line at the superhydrophobic/hydrophilic surface. Adapted with permission from Ref. [100]. Copyright 2022 American Chemical Society. (**D**) The superhydrophobic property of the material can be controlled by stretching the object. Adapted with permission from Ref. [101]. Copyright 2024 American Chemical Society.

Inspired by *Salvinia molesta* and lotus leaf, a layered gas shield film with regular micron-sized cone array was constructed by using silicone rubber (SR), graphene (GN) and SiO_2_ to achieve antifouling effect (Figure 6A) [43]. A superhydrophobic cotton fabric featuring a lotus leaf-inspired papillary microstructure was developed through the in situ growth of cross-shaped ZIF-L nanosheets combined with PDMS surface modification, exhibiting excellent wash and wear resistance (Figure 6B) [86]. An alternative approach using femtosecond laser-induced coarsening of polytetrafluoroethylene (PTFE) successfully mimicked the bubble-absorbing properties of lotus leaves. Interestingly, this surface not only exhibits superaerophilicity underwater but can also be artificially switched to superaerophobicity, and this reversible switch enables “localization trapping” of bubbles [16]. Inspired by the self-cleaning mechanism of lotus leaves, laser-etching technology was employed to fabricate bionic superhydrophobic surfaces (BSSs) with micro-convex arrays and bionic superhydrophobic/hydrophilic hybrid surfaces (BSHSs) with alternating stripes. Hydrophilic strips pinned bubbles to form continuous air plastron chains, which synergistically reduced drag resistance by 30% even at high flow velocities (4.45 m/s) (Figure 6C) [100]. The stretchable superhydrophobic structure can be manufactured by using the Digital Light Processing (DLP) 3D printing technology, and the superhydrophobic performance can be reversibly adjusted by changing the micro-column spacing through stretching according to the elastic deformation of the material (Figure 6D) [101].

### 4.4. Water Bug Notonecta glauca-Inspired Air Layer Surface

Bionic surfaces inspired by water bugs primarily consist of nanostructured hairs that replicate those of natural prototypes. In a seminal study, Kavalenka et al. [102] employed a scalable thermal stretching technique to fabricate biomimetic nano-hairs on sandblasted steel substrates, mimicking the elytra surface morphology of *N. glauca*. Their investigation revealed that these artificial nano-hairs could maintain underwater air layers with stability comparable to *Salvinia* species and superior to lotus leaf surfaces [102]. Building upon this foundation, Liu et al. [87] developed an innovative bilayer superhydrophobic surface through the synergistic combination of hydrothermal processing and electrospinning techniques. This hierarchical architecture, drawing inspiration from both *N. glauca* elytra and spider silk, demonstrated exceptional gas retention capabilities and significantly improved resistance to hydrostatic pressure [87]. Despite these advances, conventional biomimetic structures often exhibit limited air layer stability under increasing hydrostatic pressures. Addressing this challenge, Vuellers et al. [103] engineered pressure-resistant polymer nano-hairs based on *N. glauca* morphology, achieving breakthrough performance in maintaining stable air layers even under extreme hydrostatic conditions.

### 4.5. Diving Beetle-Inspired Air Layer Surface

**Figure 7:**
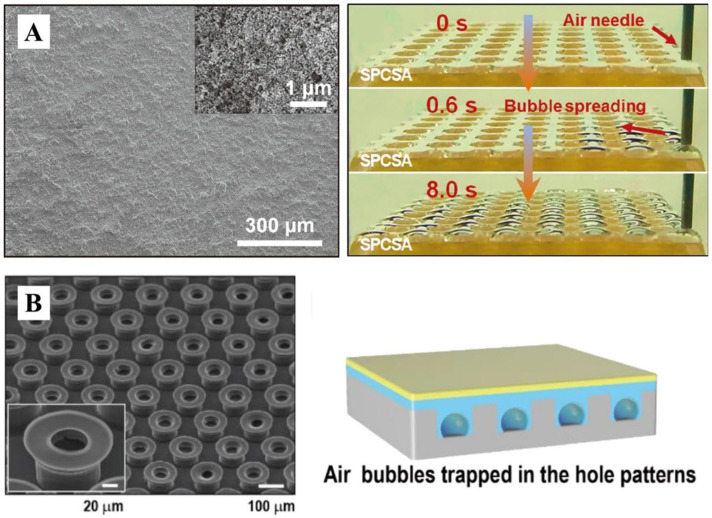
(**A**) SEM image of SPCS surface and optical image of bubbles spreading on the surface. Adapted with permission from Ref. [88]. Copyright 2024 Wiley. (**B**) Combining spherical chambers and mushroom-shaped tips to trap air bubbles. Adapted with permission from Ref. [104]. Copyright 2021 Elsevier Ltd.

Drawing inspiration from diving beetle morphology, Zhang et al. [88] developed a superhydrophobic polymethyl methacrylate circular sheet (SPCS) through laser cutting and surface coating techniques, demonstrating exceptional bubble adhesion capabilities (Figure 7A). Building upon the hair-like microstructures observed in diving beetles, Song et al. [104] engineered a novel multiscale 3D architecture integrating spherical chambers with mushroom-shaped tips. This biomimetic design enabled effective air capture within the spherical cavities (Figure 7B). Furthermore, the researchers successfully translated this structural concept into a bioadhesive material, achieving robust adhesion performance on various challenging substrates including soft, moist, and irregular biological surfaces such as skin and organs [104].

### 4.6. Water Strider-Inspired Air Layer Surface

**Figure 8:**
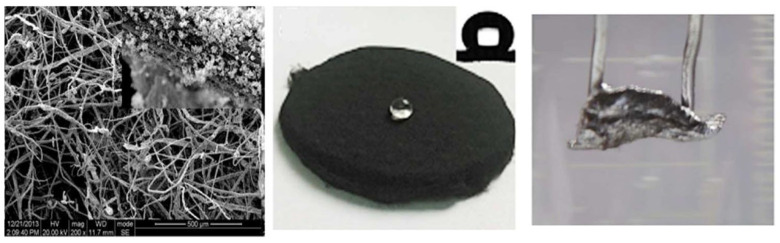
A porous membrane hybridized with cellulose fibers and Ag/Ag_2_O nanoparticles produces a specular reflection air layer underwater. Adapted with permission from Ref. [89]. Copyright 2015 Elsevier Ltd.

To simulate the characteristics of water striders walking on water, Wang et al. [89] developed a biomimetic porous membrane through in situ hybridization of bamboo cellulose fibers with Ag/Ag_2_O nanoparticles. The fabricated membrane exhibited superior hydrophobicity with a water contact angle of 140°. Notably, when submerged in water, the membrane’s surface could trap air layers sufficiently stable to produce mirror-like reflection phenomena (Figure 8) [89].

### 4.7. Rose Petals-Inspired Air Layer Surface

**Figure 9:**
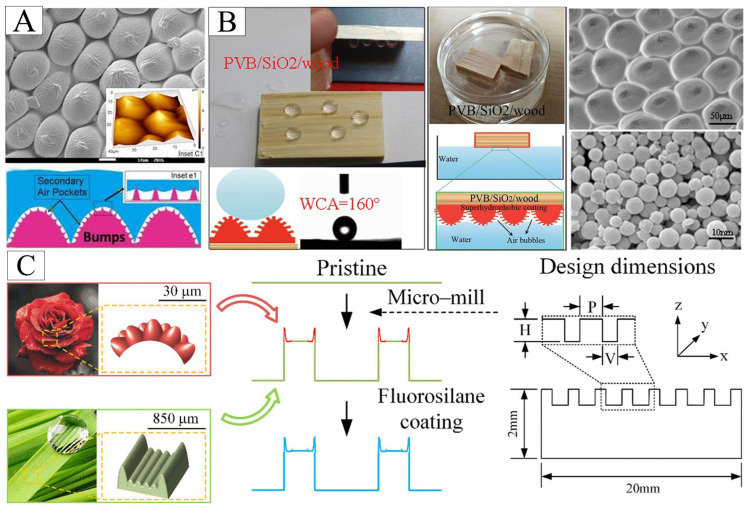
(**A**) The biomimetic surface of rose petals prepared from hydrophobic silicon particles and hydrophobic nanofibers can form air pockets under water. Adapted with permission from Ref. [106]. Copyright 2019 Elsevier Ltd. (**B**) Biomimetic PVB/SiO_2_/wood traps bubbles underwater. Adapted with permission from Ref. [107]. Copyright 2019 Springer Nature. (**C**) The rice leaf–rose petal structures coupled (RRSC) aluminum surfaces were fabricated by micro-milling. H, V, and P represent groove depth, groove width, pillar width. Adapted with permission from Ref. [90]. Copyright 2025 American Institute of Physics.

The researchers used metal deposition, photolithography and 3D printing to obtain unique structures based on the microstructure of rose petal surface. Yuan et al. [105] deposited textured silver onto a copper substrate via electroless plating, achieving rose-petal-like biomimetic structures with controlled deposition time, which exhibit both water droplet adhesion and air retention properties. Ghosh et al. [106] adopted an alternative approach, co-spraying hydrophilic silica particles with hydrophobic nanofibers to create highly hydrophobic yet strongly water-adhesive rose-petal-mimetic surfaces, observing stable air pockets within the microstructure underwater (Figure 9A). Meanwhile, Yang et al. [107] successfully fabricated superhydrophobic polyvinyl butyral PVB/SiO_2_ coatings on wood surfaces by one-step solvothermal method and nanoimprint lithography. The PVB/SiO_2_ coating has a microstructure similar to that of rose petals with a static CA of 160° in air, and is capable of trapping air bubbles underwater (Figure 9B) [107]. Bionic multifactor coupling may lead to better performance, for example, Wan et al. [90] fabricated a rice leaf-rose petal coupled structure (RRSC) aluminum surface by micro-milling, which is more hydrophobic than a single biomimetic surface (Figure 9C). The contact angle per micron of rose petal crown width has increased by 0.57°, and the runoff angle has increased by 3.34°, which can retain the air layer by hindering droplet penetration. This structural coupling approach achieves enhanced wettability performance by strategically integrating complementary surface characteristics from different natural prototypes.

### 4.8. Basilisk lizards-Inspired Air Layer Surface

**Figure 10:**
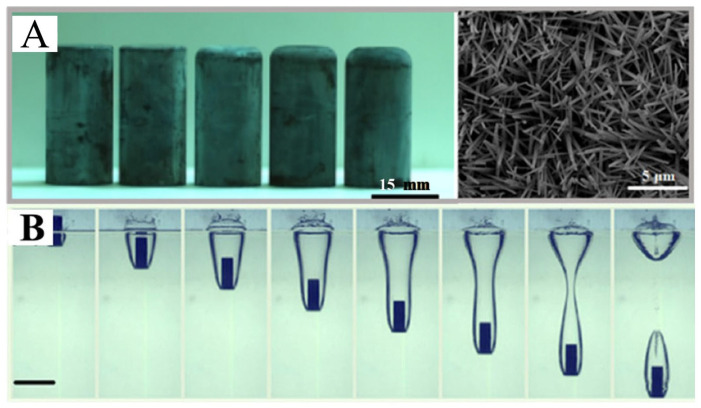
(**A**) Optical and SEM images of the bionic cylindrical cylinder. (**B**) The bionic cylindrical cylinder forms an air cavity in the water. Adapted with permission from Ref. [84]. Copyright 2021 American Chemical Society.

Inspired by the toe morphology of *Basilisk lizards*, Yao et al. [84] proposed a novel strategy for inducing and stabilizing an air–water interface through free-surface water shock using cylindrical steel impactors (Figure 10). This study demonstrated that the downward impact of the cylinder deforms the air–water interface, generating a transient cavity. By carefully controlling the dynamic shock process of the free water surface, they successfully achieved a stable underwater air–water interface was successfully achieved [84].

### 4.9. Scales-of-Carp-Inspired Air Layer Surface

**Figure 11:**
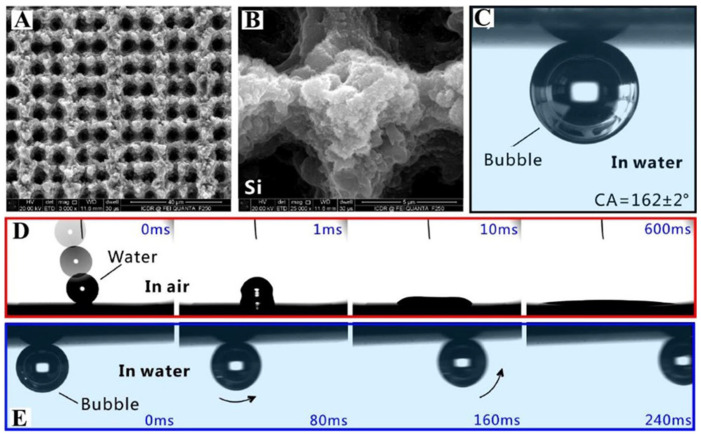
(**A**,**B**) SEM images of the silicon surface after laser ablation. (**C**) Adsorption of air bubbles in water onto the rough silicon surface. (**D**) Wetting of water droplets on rough surfaces in air. (**E**) The process of underwater bubbles rolling on a rough surface. Reprinted with permission from Ref. [55]. Copyright 2017 American Chemical Society.

Building upon the discovery of superaerophobic properties in carp scales, Yong et al. [55] employed femtosecond laser ablation to fabricate a biomimetic microstructured silicon surface (Figure 11A,B). This engineered surface demonstrates remarkable dual wettability characteristics: exhibiting superhydrophilicity in ambient conditions while transitioning to superaerophobic behavior upon water immersion. This unique wetting transition enables effective underwater air bubble trapping, as demonstrated in Figure 11C–E [55]. The surface’s ability to maintain stable air pockets stems from its precisely controlled micro/nanostructures, which mimics the natural aerophobic properties observed in carp scales while introducing enhanced functionality through laser patterning.

Modern fabrication techniques including 3D printing, photolithography, electrostatic flocking, and femtosecond laser processing enable the creation of precision-engineered surfaces capable of stable air layer retention. These advanced manufacturing methods are complemented by functional microstructures such as microcavity arrays and shape memory polymer networks, which significantly enhance air-trapping performance. While artificial surfaces may not perfectly replicate the complex hierarchical architectures found in natural systems, they offer distinct advantages in controlled environments. Current technological limitations include challenges in scalable production, elevated manufacturing costs, and process complexity. Notably, engineered air layers demonstrate superior performance under extreme hydrostatic conditions, maintaining stability for extended periods exceeding 60 days—a durability benchmark surpassing many natural counterparts.

## 5. Application of Air Layer Structure

### 5.1. Air Layer in Antifouling Treatment

#### 5.1.1. Antifouling Mechanisms

The trapped air layer serves as an eco-friendly physical antifouling barrier, effectively inhibiting marine biofouling by preventing the attachment of marine organisms [9]. This mechanism is facilitated by superhydrophobic surface micro/nanostructures that stabilize the air layer, thereby significantly reducing the effective contact area between the material surface and fouling microorganisms [108,109]. The air layer’s impermeability creates a physical blockade that prevents microbial colonization, ultimately achieving durable antifouling performance without relying on biocidal chemicals.

#### 5.1.2. Factor Affecting Antifouling of Air Layer

The antifouling efficacy of air layers is significantly influenced by air layer stability and air pocket dimensions. Research indicates that prolonged antifouling protection directly correlates with the durability of the air layer. According to Hwang et al.’s study [110] on the adhesion of *Staphylococcus aureus* on the surface of artificial air layer, it can be seen that the bacterial adhesion can be effectively reduced when the air layer exists, but the bacterial adhesion is obviously not restricted when the air layer disappears. Scardino et al. [111] immersed the synthetic material in the real marine environment for a long time and found that the long-term maintenance of the bubble layer can significantly reduce the degree of biofouling, especially for large fouling organisms. Importantly, Wu et al. [112] elucidated the size-dependent mechanism, and demonstrated that diatom adhesion was effectively inhibited on macroscopic air pockets, whereas negligible inhibition occurred on nanoscale air pockets.

#### 5.1.3. Antifouling Application

**Figure 12:**
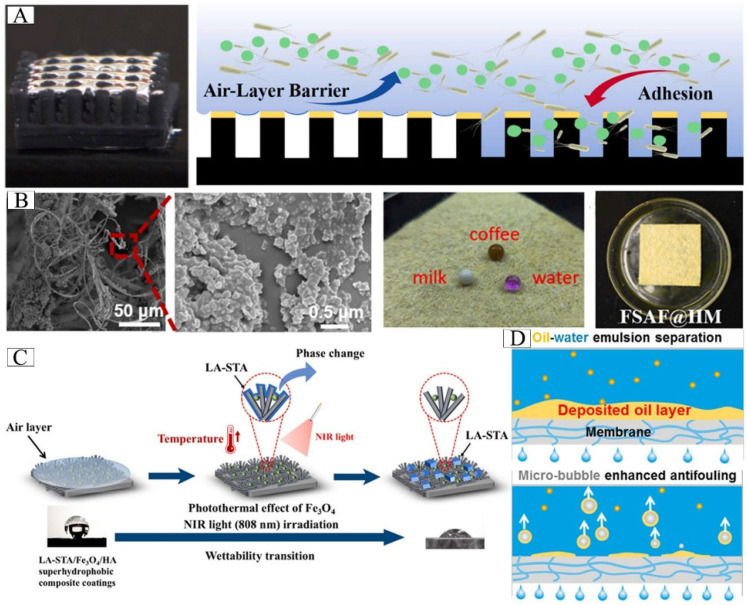
(**A**) Schematic diagram of bionic material for trapping bubble and its physical barrier for antifouling. Reprinted with permission from Ref. [9]. Copyright 2025 Elsevier Ltd. (**B**) SEM pictures of aramid fiber modified and its self-cleaning property. Reprinted with permission from Ref. [113]. Copyright 2025 Elsevier Ltd. (**C**) Wettability transition mechanism of LA-STA/Fe_3_O_4_/HA composite coatings. Adapted with permission from Ref. [114]. Copyright 2025 Elsevier Ltd. (**D**) In situ microbubbles achieve broad-spectrum and high-efficiency antifouling performance through the hydrophobic adsorption-floating migration. Adapted with permission from Ref. [116]. Copyright 2021 Elsevier Ltd.

Recent advances in air-layer-based antifouling technologies demonstrate remarkable versatility across material systems. Air layers serve as an excellent physical barrier that can effectively isolate fouling organisms from material surface without releasing toxic substances. Inspired by *Salvina* and lotus leaf, Fu et al. [43] designed an environmentally friendly silicone rubber composite, which can effectively prevent the adhesion of Gram-negative bacteria (*Paracoccus pantothenate*), Gram-positive bacteria (*Bacillus subtilis*) and algae (*Chlorella pyrenoidosa*). Sun et al. [9] developed a biomimetic surface capable of stabilizing underwater air layers, creating durable physical antifouling barriers that eliminate biocidal environmental impacts (Figure 12A). Inspired by *Salvinia*, Yang et al. [113] fabricated superhydrophobic aramid fiber surfaces exhibiting exceptional self-cleaning properties (Figure 12B). Recent research has demonstrated a variety of innovative approaches for surface modification and antifouling applications. An intelligent composite coating was developed for magnesium alloys using Fe_3_O_4_, hydroxyapatite and fatty acids (lauric acid/stearic acid), which exhibited a reversible conversion from superhydrophobicity to hydrophilicity under near-infrared irradiation, thereby inhibiting bacterial adhesion (Figure 12C) [114]. Chemical oxidation processes have been successfully applied to create microstructured oxide layers on aluminum, stainless steel, and titanium substrates, forming stable air-trapping structures with significant antifouling effects [115]. For marine applications, silicone coatings with precisely engineered bubble-generating microcavity arrays have shown velocity-dependent antifouling performance, effectively reducing adhesion of marine bacteria and diatoms under both static and dynamic conditions [96]. In addition to preventing marine biofouling, air layer technology has also been extended to the field of oil pollution control by utilizing membrane systems that leverage the hydrophobic interaction between microbubbles and oil contaminants. In environmental engineering, bubble curtain systems have been proven to be particularly effective in dredging operations. Wang et al. [116] proposed a membrane antifouling method utilizing the hydrophobic interaction between in situ-generated microbubbles and oil, as shown in Figure 12D. Engineering practice assessment shows that the bubble curtain system can effectively reduce water turbidity and demonstrates unique technical advantages [117].

### 5.2. Air Layer in Drag Reduction

#### 5.2.1. Drag Reduction Mechanisms

Drag reduction plays a crucial role in energy conservation by significantly decreasing fuel consumption. The air-layer drag reduction technique has emerged as an effective approach, where superhydrophobic surface microstructures interact with water surface tension to entrap air pockets. When submerged, these microstructures maintain a stable air–water interface that prevents direct contact between the water flow and solid surface. Because the viscosity of water and air layers is significantly lower than that of water and solid surfaces, achieving significant drag reduction effect [118].

#### 5.2.2. Factor Affecting Drag Reduction of Air Layer

The drag reduction effect of an air layer hinges on a stable gas film (Cassie state) at the solid–liquid interface, yet its efficacy is modulated by surface morphology and flow regime. In laminar flow, drag reduction primarily depends on the thickness of the trapped air layer and the fraction of gas–liquid contact area. Studies indicate that increased shear rates compress the air layer, reducing its thickness and gas coverage, thereby diminishing drag reduction [119]. While higher groove area ratios (i.e., gas–liquid interface fractions) initially enhance drag reduction by expanding the interfacial area, surpassing a critical threshold induces gas film instability that negates this benefit. In turbulent flow, sustainable drag reduction demands ultra-low surface roughness relative to the viscous sublayer thickness, coupled with highly porous textures. When surface roughness is sufficiently small, significant drag reduction persists even at high Reynolds numbers. Conversely, excessive roughness generates turbulent dissipation that overwhelms the air layer’s advantage [120]. Porosity further promotes stability by entrapping air pockets against high shear stresses. Thus, optimizing air layer thickness and maintaining groove area ratios within a stable range are key to efficient drag reduction, with turbulence demanding stricter control of both parameters.

#### 5.2.3. Drag Reduction Application

**Figure 13:**
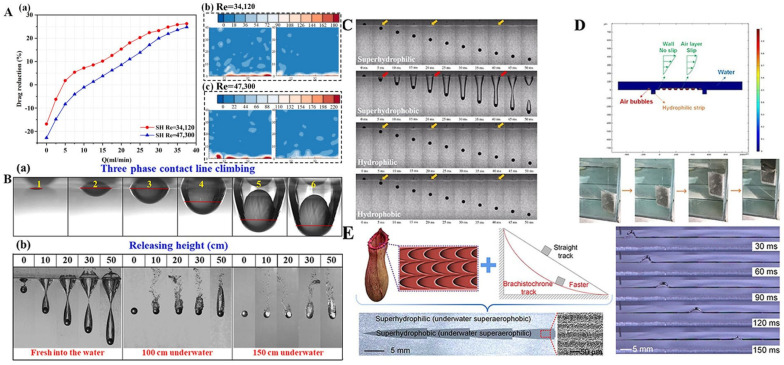
(**A**) (**a**) The relationship between flow rate Q and drags reduction rate at Re = 34,120 and Re = 47,300. (**b**) The vorticity for Re = 34,120 and (**c**) Re = 47,300 conditions without air injection and at the highest rate of drag reduction. Adapted with permission from Ref. [121]. Copyright 2024 Elsevier Ltd. (**B**) (**a**) Snapshot of the three-phase contact line (red line) climbing up the superhydrophobic steel sphere during water entry. (**b**) Cavity morphologies at various depths for 20 mm superhydrophobic spheres dropped from different heights. Adapted with permission from Ref. [123]. Copyright 2025 Elsevier Ltd. (**C**) Snapshots of spheres with the same diameter and different surface wettability after falling from the same height. Reprinted with permission from Ref. [124]. Copyright 2021 American Chemical Society. (**D**) Air bubbles mechanism generation and interface slippery analysis of 2D ASHS simulation model, and snapshots of moving continuously from underwater to overwater of ASHS. Adapted with permission from Ref. [125]. Copyright 2021 Elsevier Ltd. (**E**) The device principle and the underwater transportation behavior of bubbles. Adapted with permission from Ref. [126]. Copyright 2024 Royal Society of Chemistry.

Zhu et al. [121] maintained air layer stability through active air injection into porous steel substrates. Their analysis demonstrated that even at high flow rates, actively injecting air into superhydrophobic porous steel can maintain a stable air layer, with resistance reduced by more than 25% (Figure 13A). Leveraging the ability of hydrophobic surfaces to adsorb underwater bubbles, Gao et al. [122] applied bubble growth and detachment from the surface to reduce drag in polydimethylsiloxane (PDMS) microchannels. Zhao et al. [123] fabricated superhydrophobic steel balls with hydrophilic strips using spray modification and laser processing. Compared to fully coated spheres, localized hydrophilic patterning (positioned at or above the equator) significantly enhanced cavity stabilization and prolonged drag reduction (Figure 13B). Yao et al. [124] grew a large number of uniform micro-branch structures on the surface of copper spheres to simulate the abdomen of water spiders, and placed copper spheres with and without micro-branches underwater to study the effect of air layer on drag reduction. As shown in Figure 13C, spheres with micro-branches can reduce drag by more than 90%. Inspired by the cylindrical toes of *basilisk lizards*, Yao et al. [84] prepared hydrophilic, hydrophobic, superhydrophilic and superhydrophobic cylinders, simulating the air–water interface formed by the toes of *Basilisk lizards* underwater, and studying their drag reduction ability. These results indicated that the shape and volume of the cavity have little correlation with the wettability of the cylinder surface, but are related to the release height and geometry of the cylinder. In addition, altering the cylinder geometry can significantly improve the drag reduction efficiency. To compensate for the poor drag reduction effect caused by the instability of air layer, Rong et al. [125] inspired by the asymmetric micro/nanostructured arrays of fish scales, proposed a simple manufacturing method for anisotropic superhydrophobic/hydrophilic surfaces (ASHS). This approach strengthens the air/water/solid three-phase contact line and captures surrounding bubbles, thereby reducing drag under high-velocity flow conditions, as illustrated in Figure 13D. In order to obtain an environmentally friendly drag reduction method, inspired by Nepenthes lip and the brachistochrone curve, Yan et al. [126] developed a device that can capture underwater bubbles and realize directional spontaneous delivery. The speed of bubble transportation of this device is 444 mm/s, and the drag reduction rate reaches 27% (Figure 13E).

In reality, marine antifouling and drag reduction are fundamentally interdependent phenomena. Ship biofouling not only accelerates hull corrosion but also substantially increases hydrodynamic resistance, resulting in significant fuel overconsumption. Consequently, implementing integrated antifouling and drag-reduction strategies can effectively mitigate both biological colonization and frictional resistance, thereby addressing the dual concerns of invasive species accumulation and energy inefficiency. The convergence of these technologies constitutes a critical solution for sustainable maritime development. By simultaneously resolving biofouling-induced corrosion and hydrodynamic resistance, the synergistic effects extend hull service life while complying with increasingly stringent environmental regulations, making this approach an essential strategic response to the economic and ecological pressures confronting modern shipping industries.

### 5.3. Other Applications of Air Layers

#### 5.3.1. Thermal Insulation and Anti-Freezing

The core goal of thermal insulation is to inhibit heat transfer via conduction, convection, and radiation. Air layers leverage the inherent low thermal conductivity of air and suppress convective heat transfer to achieve thermal insulation. Therefore, using an air layer with both thermal insulation and waterproof properties has become an effective way to solve the icing problem. In maritime operations, surface icing presents a critical challenge that triggers a cascade of detrimental effects, including escalated energy consumption, diminished operational efficiency, compromised structural stability, accelerated material degradation, resulting in major safety hazards. To address this multifaceted issue, superhydrophobic and thermally insulating air layers have emerged as a promising solution. Zhu et al. [37] achieved excellent anti-icing performance by injecting gas into micro-nano porous steel, forming a static air cushion and dynamic “air armor” that inhibited condensation and ice nucleus formation, blocked heat transfer paths, reduced ice adhesion strength, and delayed ice formation.

#### 5.3.2. Electrochemical Energy Conversion

The air layer is not a direct component of the electrochemical core reaction, but it is closely related to the electrochemical process by affecting key aspects such as the interface state of the electrochemical system, material transport, and reaction efficiency. Electrochemical reactions (such as redox, hydrogen/oxygen evolution) rely on effective contact at the electrode-electrolyte interface, as well as efficient transport of reactants/products at the interface. However, the air layer can disrupt or alter these two core conditions. Specifically, the air layer can damage the electrode-electrolyte contact, leading to “interface deactivation,” hinder material transport, and reduce reaction efficiency.

However, air layers are not inherently detrimental—when rationally designed, they can be actively harnessed to optimize electrochemical performance. For instance, air electrodes in zinc-air batteries use a “porous structure” to retain a trace air layer (as an O_2_ transport channel) while ensuring electrolyte access to active sites. For corrosion protection, air layer can isolate metals from corrosive electrolytes (such as seawater) to mitigate electrochemical corrosion.

Inspired by gas-management strategies of aquatic organisms, biomimetic electrodes are being developed to optimize these processes. For instance, superhydrophobic or superaerophobic surfaces can facilitate the rapid detachment of bubbles, thereby preventing electrode fouling and mass transfer limitations. Conversely, trapped air layers or engineered gas-trapping interfaces can be utilized to create a stable gas–liquid–solid interface, thereby enhancing the local concentration of gaseous reactants such as nitrogen (N_2_). Inspired by the hierarchical structure of aquatic organisms, Li et al. developed an electrode with simultaneous superaerophilic and superhydrophobic properties that effectively enrich N_2_ molecules at the triple-phase boundary, thereby improving the efficiency and selectivity of electrocatalytic ammonia synthesis [127]. This demonstrates that the principles of bubble capture and management learned from nature can be translated into advanced applications in renewable energy and chemical synthesis.

## 6. Conclusions and Outlook

This review has summarized various classic biological cases of air layer retention and the corresponding advanced biomimetic fabrication techniques. We have discussed how these bioinspired air layers serve as solutions for antifouling and drag reduction, highlighting the critical roles of surface micro/nanostructures and wettability. However, there are still significant challenges in transitioning from laboratory prototypes to practical applications. Future research should focus on addressing the following critical issues.

### 6.1. Scalability and Cost-Effective Manufacturing

Most current biomimetic fabrication methods (such as femtosecond laser processing and lithography technology) have high accuracy, but suffer from low throughput and high cost, making them unsuitable for large-scale applications such as ship hulls. Future efforts should therefore focus on developing scalable, cost-effective, and sustainable techniques that enable the fabrication of robust hierarchical structures on large and curved surfaces.

### 6.2. Long-Term Stability

Laboratory tests are typically conducted in idealized and quiescent water. The real environments involve hydrostatic pressure, flowing water (high shear stress), temperature fluctuations, and biological attack, all of which can damage the air layer. This requires designing a surface with enhanced pressure resistance by optimizing the re-entrant structures and elastic materials. Furthermore, exploring active regeneration systems that can repair damaged structures or replenish the air layer, perhaps by integrating microfluidic networks or exploiting cavitation effects to generate gas in situ, represents a crucial direction.

### 6.3. Dynamic and Responsive Systems

Current biomimetic surfaces predominantly exhibit passive and static characteristics, with their performance being inherently fixed after fabrication. A promising avenue is to develop smart, responsive interfaces whose wettability and morphology can change in response to external stimuli (such as pH, temperature, light, electric field). Such adaptive systems could maintain optimal performance across different working conditions, enabling on-demand activation of antifouling or drag reduction properties. Such intelligent response will significantly enhance long-term operational efficiency and sustainability by optimizing the utilization of energy and materials [128].

### 6.4. Multifunctional Integrated Design

Current research typically focuses on single functionalities (e.g., drag reduction), whereas practical applications require multi-functionality. Therefore, it is crucial to endow air layer technology with other functions, such as corrosion resistance, sensing (monitoring biofilm formation or air layer state), and energy harvesting. For instance, the hull must simultaneously achieve drag reduction, antifouling, and corrosion resistance ability, while underwater monitoring equipment could benefit from combining drag reduction with biofilm sensing. These examples emphasize that the design principles must shift from merely focusing on the surface to adopting a holistic system-level perspective.

### 6.5. Quantitative Prediction and Inverse Design

Current bioinspired design processes are primarily driven by inspiration from nature and remain largely empirical. We lack reliable models to precisely predict the performance of a proposed structure. Leveraging multiphysics simulations and machine learning to create models to accurately predict the stability and performance under complex conditions will be a key priority. This will enable inverse design—where desired performance parameters are specified, allowing algorithms to generate optimal surface structures [129].

## Data Availability

Data requests can be emailed to the corresponding author’s email address.
